# Histopathological Analysis of PEEK Wear Particle Effects on the Synovial Tissue of Patients

**DOI:** 10.1155/2016/2198914

**Published:** 2016-08-16

**Authors:** A. C. Paulus, S. Haßelt, V. Jansson, A. Giurea, H. Neuhaus, T. M. Grupp, S. Utzschneider

**Affiliations:** ^1^Department of Orthopaedic Surgery, University Hospital of Munich (LMU), Campus Großhadern, Marchioninistraße 15, 81377 Munich, Germany; ^2^Department of Orthopaedics, Vienna General Hospital, Medical University of Vienna, Waehringer Guertel 18-20, 1090 Vienna, Austria; ^3^Department of Traumatology and Orthopaedics, St. Vincenz Hospital, Am Stein 24, 58706 Menden, Germany; ^4^Aesculap AG Research & Development, Aesculap-Platz 1, 78532 Tuttlingen, Germany

## Abstract

*Introduction*. Increasing interest developed in the use of carbon-fiber-reinforced-poly-ether-ether-ketones (CFR-PEEK) as an alternative bearing material in knee arthroplasty. The effects of CFR-PEEK wear in* in vitro* and animal studies are controversially discussed, as there are no data available concerning human tissue. The aim of this study was to analyze human tissue containing CFR-PEEK as well as UHMWPE wear debris. The authors hypothesized no difference between the used biomaterials.* Methods and Materials*. In 10 patients during knee revision surgery of a rotating-hinge-knee-implant-design, synovial tissue samples were achieved (tibial inserts: UHMWPE; bushings and flanges: CFR-PEEK). One additional patient received revision surgery without any PEEK components as a control. The tissue was paraffin-embedded, sliced into 2 *μ*m thick sections, and stained with hematoxylin and eosin in a standard process. A modified panoptical staining was also done.* Results*. A “wear-type” reaction was seen in the testing and the control group. In all samples, the UHMWPE particles were scattered in the tissue or incorporated in giant cells. CFR-PEEK particles were seen as conglomerates and only could be found next to vessels. CFR-PEEK particles showed no giant-cell reactions. In conclusion, the hypothesis has to be rejected. UHMWPE and PEEK showed a different scatter-behavior in human synovial tissue.

## 1. Introduction

Aseptic loosening after total joint arthroplasty is still the main reason for failure of the prostheses and subsequently for revision surgery [1, 2]. The complex mechanism of the aseptic loosening is to this day not understood in detail, but wear particles play an important role in this process [1–3]. Several* in vitro* studies could proof the influence of material, particle number, size, and shape, and the extent of an inflammatory process, which finally leads to an osteolysis [4–6]. Ultra-high-molecular-weight-polyethylene (UHMWPE) is still the bearing material of choice especially in knee arthroplasty, but in regard to the development of a significant amount of wear particles inducing aseptic loosening, there exists a growing demand for alternative bearing materials [7, 8]. Lately, increasing interest developed in the use of carbon-fiber-reinforced-poly-ether-ether-ketones (CFR-PEEK) [7, 9, 10]. PEEK became more and more interesting for the use as biomaterial in trauma and orthopaedic applications, as it has already been successfully employed in spinal surgery [11, 12]. While there is a lack of data concerning the effects of CFR-PEEK particles on human tissue, the effects of this wear debris in* in vitro* and in animal studies are controversially discussed [7, 9]. In a previous study, PEEK particles, generated in a knee simulator testing unicondylar knee replacements, seemed to provoke an elevated biological reaction* in vivo* in a balb/c mice model [7].

Nevertheless, it seems impossible to draw conclusions from a mouse model on humans. In this context, it was the aim of this study to investigate the histologic effect of CFR-PEEK and UHMWPE wear particles on human synovial tissue. For this reason, synovial tissue, achieved from revision surgery of total knee prostheses containing CFR-PEEK as well as UHMWPE components, was investigated histologically.

The authors hypothesized no different findings between the used biomaterials because of similar size parameters of the wear particles in a prior knee simulator study of this implant [13].

## 2. Materials and Methods

Revision surgery in 10 patients with a rotating-hinge-knee-implant-design (Enduro®, Aesculap, Germany) was performed (mean age 71.3 ± 10.7 a.; 8 patients were female and 2 were male). During these operations, synovial periprosthetic tissue samples (test group) were achieved. The implant survival until revision surgery was 22 months (2.5 min.–48 max.). Reasons for revision surgery were aseptic loosening, dislocation of the tibial stem, and a patella fracture.

The tibial inserts of this knee implant design were made from UHMWPE (GUR 1020), whereas the bushings and flanges are made from CFR-PEEK containing 30% polyacrylonitrile (PAN) based carbon fibers (PEEK-Optima LT1, Invibio Ltd., Thornton-Cleveleys, UK). In a prior* in vitro* test, most of the released CFR-PEEK particles showed in a scanning electron microscope analysis a size range between 0.1 and 2 *μ*m [13] (Table 1).

For a control, periprosthetic tissue samples were gained during revision surgery of one patient (control group). The implant contained no PEEK components; the articulating surfaces were invariably made from conventional UHMWPE.

The tissue was fixed with 4% paraformaldehyde, embedded in paraffin, and sliced into 2 *μ*m thick sections stained with hematoxylin and eosin in a standard process. A modified panoptical staining (preincubation in propylenglycol; >3 h; 35°C) was also performed, in order to mark the wear particles by staining them turquoise.

All work was conducted in accordance with the Declaration of Helsinki (1964). The study was approved by the ethics committee of the local university.

## 3. Results

**Figure 1:**
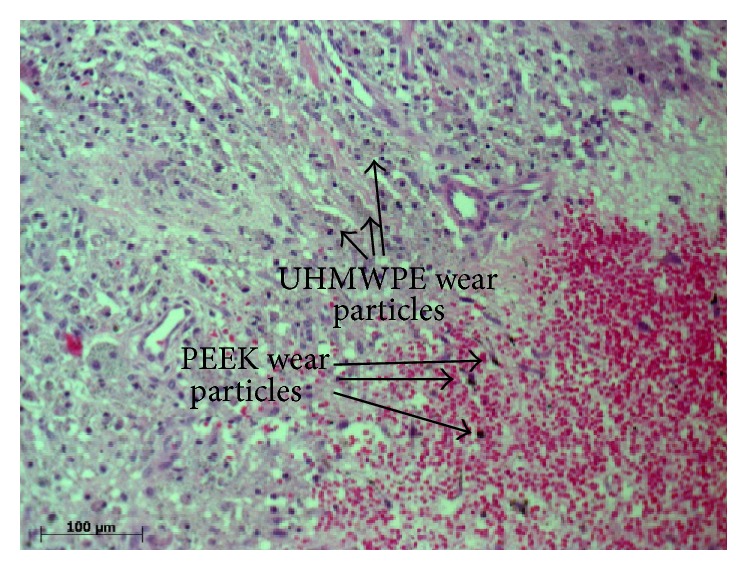
Histologic overview representing a “wear-type” [14, 15] reaction showing UHMWPE and CFR-PEEK particles stained with hematoxylin and eosin.

Throughout all samples, histologically a typical “wear-type” reaction was seen in the test as well as in the control group (Figure 1) [14, 15]. These findings were expectedly similar as described for other biomaterials in the common literature regarding wear particle associated biological reactions* in vivo*.

**Figure 2:**
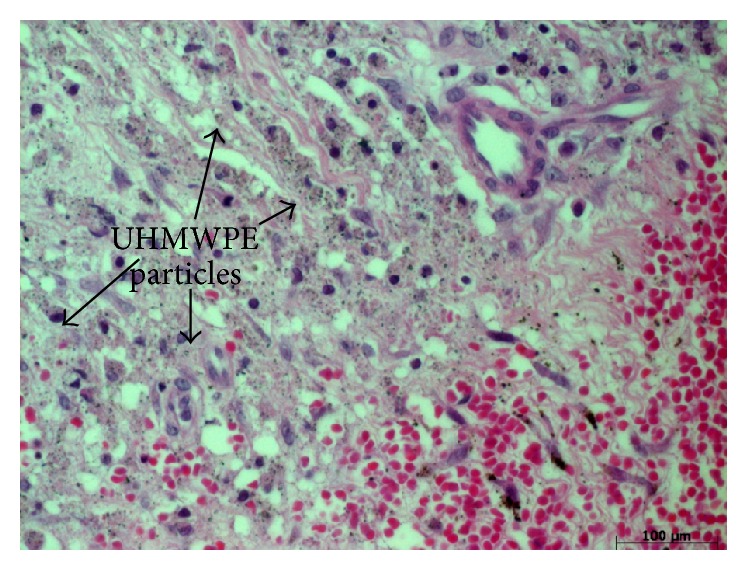
UHMWPE particles were randomly scattered in the periprosthetic tissue. The sample is stained with hematoxylin and eosin.

**Figure 3:**
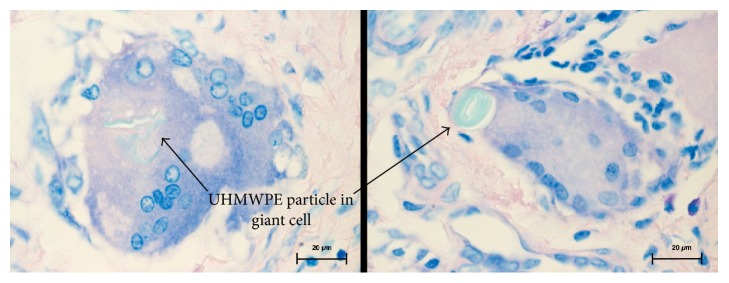
Large UHMWPE particles were incorporated in giant cells. Hematoxylin and eosin staining, 1000x magnified.

**Figure 4:**
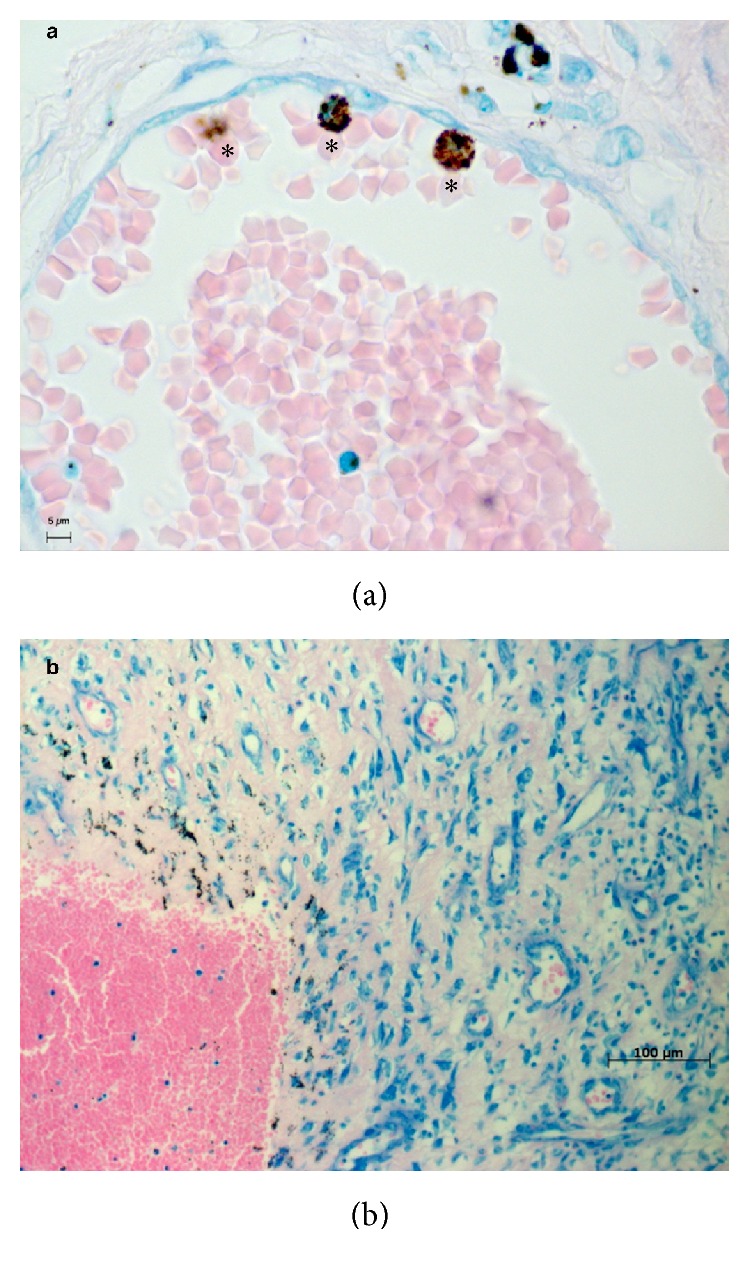
CFR-PEEK particles only could be found in or next to vessels. Giemsa staining. *∗* = in macrophages incorporated PEEK particle conglomerates.

Without exception, the UHMWPE particles in all samples of the test group were scattered in the tissue similar to the control (Figures 1 and 2). Larger UHWMPE particles were incorporated in giant cells (Figure 3). In contrast, the CFR-PEEK particles were not randomly scattered in the periprosthetic tissue but located only as conglomerates. In addition, these conglomerates have been found exclusively near to or in vessels (Figure 4). Furthermore, CFR-PEEK particles were incorporated by macrophages (Figure 4), but no giant-cell reactions could be seen. This characteristic applies to all size ranges of the CFR-PEEK particles.

## 4. Discussion

The initial hypothesis has to be rejected. A completely different behavior between the UHMWPE and the CFR-PEEK particles in human tissue could be found.

The biological activity of wear particles plays an important role in the pathway of the aseptic loosening process and therefore is a key factor for the survival rate of implants used for joint arthroplasty [1, 2]. There are several* in vitro* studies that examine the effect of UHMWPE wear particles on different cells, mainly macrophages [4, 6, 16]. In contrast, there are only a few studies concerning the biologic activity of PEEK [17–19]. A recent study compared CFR-PEEK pitch to PEEK-PAN and UHMWPE particles in a murine model and found rather negative effects for the PEEK variants [7]. But still there are data missing that show the biologic effects of PEEK particles in human tissue.

Thus, this is the first study that examines periprosthetic human synovial tissue from patients who underwent revision surgery. To the knowledge of the authors, there are no comparable data in the common literature.

In order to allow the comparison of PEEK and UHMWPE particles in each sample, patients with the Enduro knee system (Aesculap, Germany) were chosen, as this system uses UHMWPE as common bearing material and PEEK for the bushings and flanges in one system. To reduce prosthesis-dependent side effects, only tissue samples from this type of prosthesis were accepted for the test group. Therefore, the sample size was reduced to overall 10 patients. For a control, one patient was selected with a common knee revision system without any PEEK components. The absence of PEEK was the only desired control parameter; thus, it was not necessary to include more patients to the control group.

The results are very homogenous and conclusive, as the proven facts are verifiable in all tested samples: UHMWPE particles are scattered randomly throughout the whole tissue sample without any detectable conglomerates. In addition, small UHMWPE particles are phagocytized and larger particles are incorporated by giant cells. This phenomenon is well described in the common literature [1, 2, 20]. But in contrast, the PEEK particles of all size ranges were incorporated by macrophages; giant-cell reactions could not be seen at all. And interestingly, the PEEK particles are only findable in conglomerates; solitary particles were not detectable. These conglomerates were findable heaped next to or even in vessels that lie in the synovial membrane. It has to be stated that giant-cell reactions around UHMWPE particles were detected rarely, so it cannot be concluded that giant-cell reactions around PEEK particles do not exist, as they were not seen in the analyzed slices.

From this point it is nearly impossible to forecast an eventual biologic activity, especially as the data in the literature concerning the activity of PEEK particles are very controversial. In a recent study, the biological effects of PEEK compared to UHMWPE were analyzed using intravital fluorescence microscopy and a histological evaluation [21]. The authors could not detect any differences between UHMWPE and PEEK [21]. Later on, in an immunohistochemical study with two PEEK varieties, the data showed that the wear particles of CFR-PEEK pitch provoked a significantly more intense inflammatory reaction in the articular cartilage and the bone marrow than the used control group and UHMWPE wear particles [7]. The CFR-PEEK-PAN wear particles induced a higher cytokine release in the bone marrow and synovial membrane [7]. Compared to the actual study, the PEEK varieties elicited biological activity in certain tissue areas [7]. This might be a consequence of the conglomeration behavior next to certain anatomical structures, which could be found in this study. Summarizing the results of these two consecutive studies [7, 21], the primary results seem to be antithetic. In detail, complementary testing was necessary to prove the complex effects of PEEK particles* in vivo*. In addition, these mice test-specific results cannot be transferred to humans directly.

Howling et al., for instance, did not find cytotoxic effects of CFR-PEEK wear particles* in vitro* [17]. In comparison to CoCr particles, there was less cytotoxicity on fibroblasts and monocytic cells [17]. Morrison et al. examined the effects of CFR-PEEK particles on fibroblasts and osteoblasts [18]. They did not describe any cytotoxic effects [18]. Furthermore, Rivard et al. tested the biocompatibility of CFR-PEEK in an* in vivo* rabbit model and noticed that the particles are harmless to the spinal cord of the animals [22]. Jockisch et al. found* in vivo* a foreign body reaction to CFR-PEEK plates that were implanted into rabbit muscles, but there was no difference compared to an UHMWPE implant [19]. Moreover, there was an* in vivo* study in rats comparing the effects of CFR-PEEK particles to polyethylene particles that were injected into a prepared pouch. The PEEK group seemed to be histologically less inflamed, but there was no significant difference [23].

These studies support the biocompatibility of CFR-PEEK and suggest a comparable biological activity of CFR-PEEK and other implant devices like UHMWPE. Reflecting the noticeable results of the present study, the controversial discussion about PEEK as an alternative bearing material in arthroplasty has to go on. The migration behavior of the CFR-PEEK particles in the synovial tissue remains unclear. Concerning the mechanism of migration or transport of wear particles, there exist different theories [24–27], but there is certainly need for further investigation. Overall, it can be assumed that the adjacency of the PEEK particles to vessels might lead to a vessel-bound transport mechanism. In this situation, the particles can be actively transported to adjacent tissue such as the femoral or tibial bone along an interface membrane or even lead to further systemic reactions. On the other hand, it has to be discussed if vessel-bound transported particles might decrease the local biologic reaction. Further possible reactions are definitely throughout hypothetic and thus further conclusions cannot be drawn based on the present findings.

Overall, these controversial effects of PEEK with a huge amount of open questions definitely need more investigation, especially as now a complete different migration and agglomeration behavior of CFR-PEEK compared to conventional UHMWPE could be proven. In particular, the particle surface texture as well as the surface charge of the UHMWPE and CFR-PEEK particles as a possible factor for this specific particle migration behavior has to be taken into account. And it remains unclear whether the UHMWPE or the PEEK particle migration behavior is accompanied with more negative biologic reactions.

As a limitation of the study, the low number of the tissue samples has to be named, even if the results are quite clear. A statistical analysis was not performed due to the descriptive study design, as there was no quantitative evaluation of the microscopic results.

In this study setup, CFR-PEEK was not the bearing material. Therefore, different wear mechanisms lead to the present CFR-PEEK wear debris. These mechanisms differ from the actual bearing situation of the UHMWPE insert. But in a prestudy, a comparable size range of the found particles was found, so the wear producing factors were neglected. It has to be mentioned that another wear particle analysis of the tissue bound particles was not performed in order to preserve as much tissue as possible for histologic analysis. Different size and shape parameters of the wear particles compared to the preexisting wear simulator study are thoroughly possible.

Additional immunohistochemical analyses are necessary and will be subject of following projects. Further investigations on the adjacent bone and the interface membrane might support the present findings.

## 5. Conclusion

This is the first study that compares CFR-PEEK and UHMWPE particles in human tissue. Interestingly, a complete different agglomeration behavior of UHMWPE and PEEK particles has been found in human synovial tissue. In addition, large PEEK particles are not incorporated by giant cells, as it is common with large UHMWPE particles. This aspect needs further investigation concerning the cytokine expression and also the surface texture of particles.

## Figures and Tables

**Table 1:** Size and shape parameters of the UHMWPE and CFR-PEEK particles of a prior *in vitro* simulator based study [13].

Material	Aspect ratio (mean)	Roundness (mean)	Form factor (mean)	Size (mean diameter) Original (*µ*m)
	Original	Original	Original	
UHMWPE	1,77	0,54	0,56	0.52 ± 0.67
CFR-PEEK PAN	1,65	0,62	0,6	0.96 ± 1.76
